# Supplementary material for: Male Differentiation in the Marine Copepod Oithona nana Reveals the Development of a New Nervous Ganglion and Lin12-Notch-Repeat Protein-Associated Proteolysis
Source: Biology (Basel). 2021 Jul 13;10(7):657. doi: 10.3390/biology10070657 (PMC8301441; doi:10.3390/biology10070657)

Male differentiation in the marine copepod *Oithona nana* reveals the development of a new nervous ganglion linked to Lin12-Notch-Repeat protein-associated proteolysis

Kevin Sugier[1], Romuald Laso-Jadart[1] Soheib Kerbache[1], Jos Kafer[4], Majda Arif[1], Laurie Bertrand[2], Karine Labadie[2], Nathalie Martins[2], Celine Orvain[2], Emmanuelle Petit[2], Patrick Wincker[1], Jean-Louis Jamet[3], Adriana Alberti[2] and Mohammed-Amin Madoui[1]

1. Génomique Métabolique, Genoscope, Institut François Jacob, CEA, CNRS, Univ Evry, Université Paris-Saclay, Evry, France
2. Commissariat à l'Energie Atomique (CEA), Institut François Jacob, Genoscope, Evry, France
3. Université de Toulon, Aix-Marseille Université, CNRS/INSU/IRD, Mediterranean Institute of Oceanography MIO UMR 7294, CS 60584, 83041 Toulon cedex 9, France
4. Laboratoire de Biométrie et Biologie Evolutive, Université Lyon 1, CNRS UMR 5558, France

Supplementary data

Supplementary Notes S1: **Transcriptomic data.** 3

Supplementary Notes S2: **Transcriptomic data quality.** 4

Supplementary Notes S3: **Experimental design of the protein interaction (PPI) analysis.** . 5

Supplementary Notes S4: P**rimers used for PCR amplification** 6

Supplementary Notes S**5**. **Gene annotation of the sex-determination system associated genes of *Oithona nana*** 8

Supplementary Notes S6: **Structure and localisation of the Oithona nana LDPs** 8

Supplementary Notes S7: **Functional annotation of O. *nana* genes over-expressed in male.** 9

Supplementary Notes S8: **Phylogenetic tree of On_IGFBP7.** 11

Supplementary Notes S1: **Transcriptomic data.**

RNA-seq quality metrics of the 20 samples. One nauplii sample was discarded after MA plots pairwise comparisons analysis (see Sup note 2).

Accession Numbers (PRJEB34229)		Biological replicate	# Seq clean	# Base clean	Average size of merged reads (base)
ERX3525067	Male	1	17,539,769	4,726,772,098	155
ERX3525068		2	13,355,099	3,594,123,134	155
ERX3525069		3	13,327,828	3,697,975,710	164
ERX3525070		4	10,181,747	2,833,631,554	164
ERX3525072	Female	1	13,443,737	3,784,911,561	169
ERX3525073		2	17,565,613	4,945,908,456	170
ERX3525074		3	15,129,175	4,254,430,406	170
ERX3525071		4	13,152,019	3,685,066,788	167
ERX3525076	Copepodid	1	9,737,697	2,683,086,720	159
ERX3525077		2	10,954,752	3,090,141,346	170
ERX3525078		3	13,385,311	3,732,623,083	166
ERX3525075		4	17,758,141	4,731,918,511	152
ERX3525079	Nauplius	1	14,280,023	3,868,981,343	158
ERX3525080		2	13,034,144	3,546,435,298	159
ERX3525081		3	11,718,354	3,291,309,412	169
ERX3525082	Egg	1	16,670,807	4,457,103,808	153
ERX3525083		2	14,351,720	3,906,557,216	159
ERX3525084		3	12,333,551	3,284,934,971	152
ERX3525085		4	17,427,615	4,468,263,185	142
ERX3525086		5	16,156,218	4,271,517,322	150

Supplementary Notes S2: **Transcriptomic data quality.**

MA plots pairwise comparisons between the five developmental stages. One nauplius sampled showing a biased read count distribution was discarded.

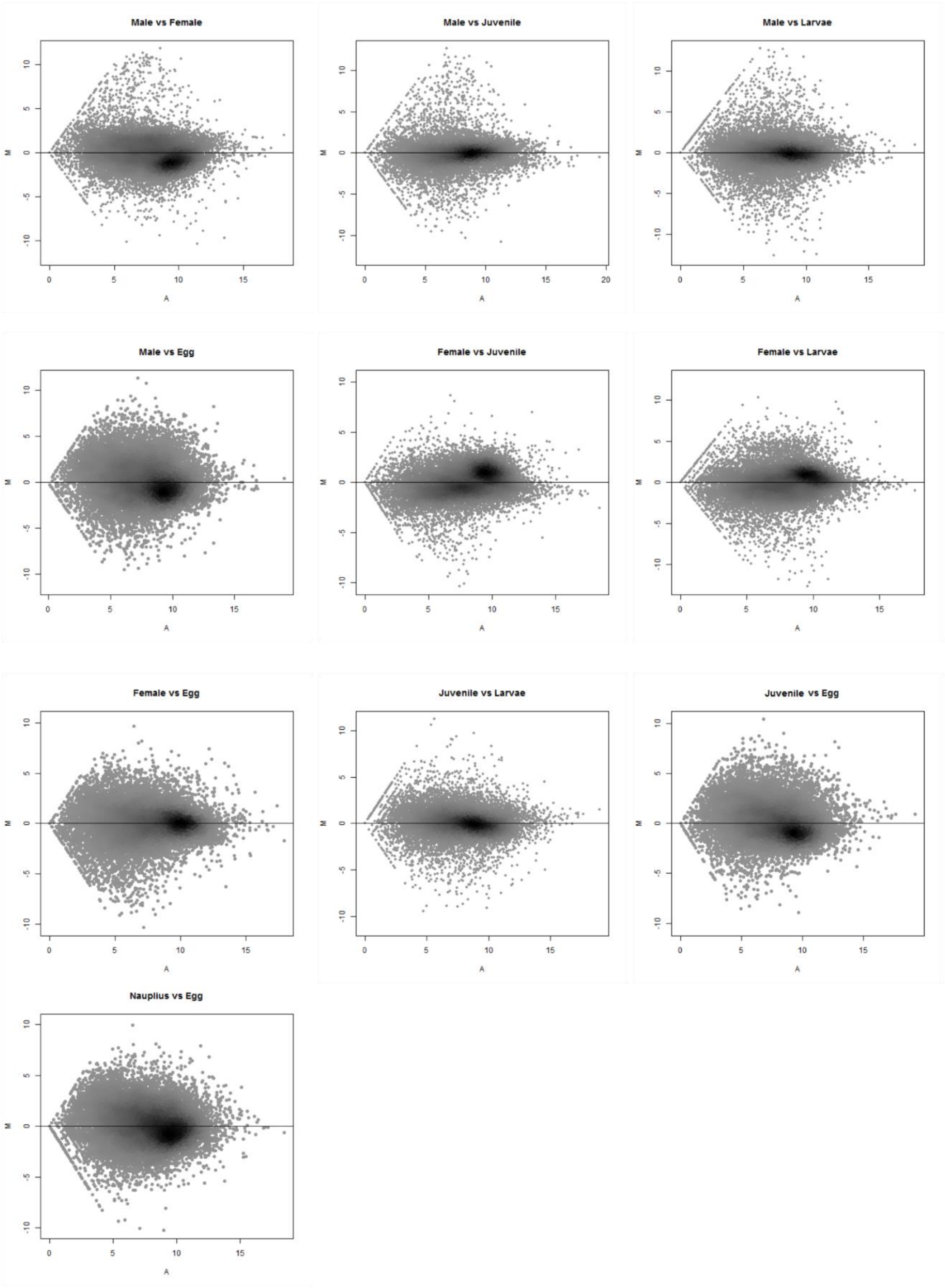

Supplementary Notes S3: **Experimental design of the protein interaction (PPI) analysis.**

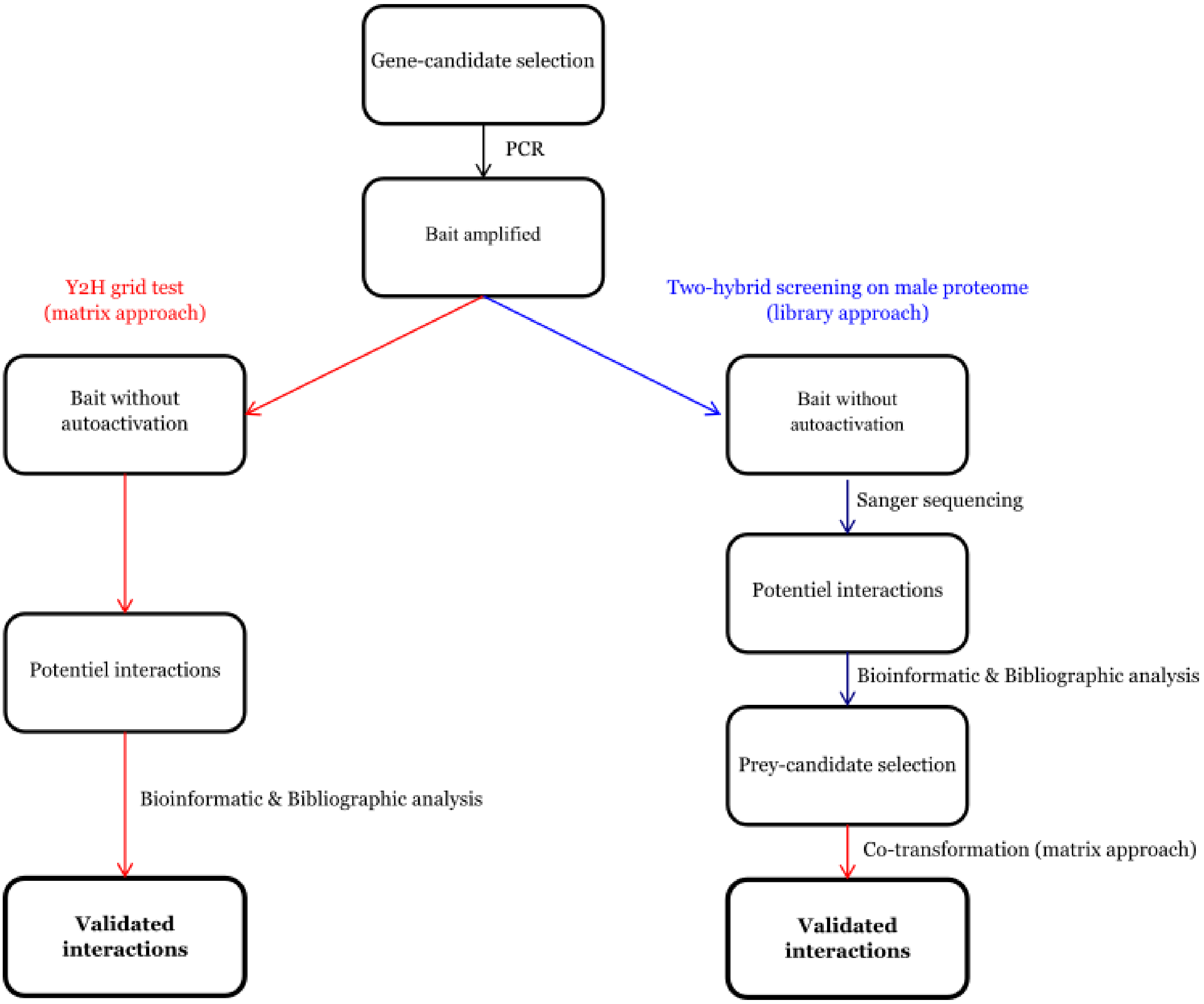

Supplementary Notes S4: P**rimers used for PCR amplification.**

The first table contains the primers used for bait amplification; the second contains the prey ones.

Gene	Elongation direction	Primer
On_LDPG1	Forward	ATGGCCATGGAGGCCGAATTCCTTCCAGGATTTGTTGGAAACTTG
	Reverse	CCGCTGCAGGTCGACGGATCCGCGAATACATTGGCAAATGGTACA
On_LDPG2	Forward	ATGGCCATGGAGGCCGAATTCCCCTATTGCCAAGCCTGTGGT
	Reverse	CCGCTGCAGGTCGACGGATCCGGTTTGAGGATACCACCGATAATC
On_IGFBP7	Forward	ATGGCCATGGAGGCCGAATTCCAACGTCGTCCACATGCCCCAAAT
	Reverse	CCGCTGCAGGTCGACGGATCCCAATTCTTTACTATAAACGCCCAC
4.25.1	Forward	ATGGCCATGGAGGCCGAATTCACTAAAATGATTCTATCAGATCGC
	Reverse	CCGCTGCAGGTCGACGGATCCTTCTTCTAGAATGATTTTATCATT
19.110.1	Forward	ATGGCCATGGAGGCCGAATTCATGTCAGTATCATATTTTAAAATC
	Reverse	CCGCTGCAGGTCGACGGATCCGCAGTCTAAACAATGATCAATTAC
49.77.3	Forward	ATGGCCATGGAGGCCGAATTCCCACAATACTTTCAACTTGGAAAC
	Reverse	CCGCTGCAGGTCGACGGATCCGAACTGGAAAATGTGGGGAATGAG
45.413.1	Forward	ATGGCCATGGAGGCCGAATTCTGTGTAGGCTGCGGAGAATACCCA
	Reverse	CCGCTGCAGGTCGACGGATCCTTGACCGGATGTCGGAGGTGGAGG
2085.199.1	Forward	ATGGCCATGGAGGCCGAATTCTTAACCACCACCCCTATTGATCTA
	Reverse	CCGCTGCAGGTCGACGGATCCCCGTGGCAATGTGCTAAATTGAGA
12.180.1	Forward	ATGGCCATGGAGGCCGAATTCTTGCCTTTCACTGTTTCCGACAAT
	Reverse	CCGCTGCAGGTCGACGGATCCCCGTGGCAATGTGCTAAATTGAGA
94.274.1	Forward	ATGGCCATGGAGGCCGAATTCGATGATGGAGGATGTGCTTTTAGG
	Reverse	CCGCTGCAGGTCGACGGATCCAGATGATCCTGTAGTGGTTGCGGT
1921.111.1	Forward	ATGGCCATGGAGGCCGAATTCGTACCCGAGTTTCAATTTGATGGA
	Reverse	CCGCTGCAGGTCGACGGATCCATCAGGATGAAAGGTGCAGTCTTG

Gene	Elongation direction	Primer
On_LDPG1	Forward	GCCATGGAGGCCAGTGAATTCCTTCCAGGATTTGTTGGAAACTTG
	Reverse	CAGCTCGAGCTCGATGGATCCGCGAATACATTGGCAAATGGTACA
On_LDPG2	Forward	GCCATGGAGGCCAGTGAATTCTTTGAACTTTGCTTTTATGATGGA
	Reverse	CAGCTCGAGCTCGATGGATCCGCAGTCTAAACAATGATCAATTAC
On_IGFBP7	Forward	GCCATGGAGGCCAGTGAATTCCAACGTCGTCCACATGCCCCAAAT
	Reverse	CAGCTCGAGCTCGATGGATCCCAATTCTTTACTATAAACGCCCAC
4.25.1	Forward	GCCATGGAGGCCAGTGAATTCACTAAAATGATTCTATCAGATCGC
	Reverse	CAGCTCGAGCTCGATGGATCCTTCTTCTAGAATGATTTTATCATT
19.110.1	Forward	GCCATGGAGGCCAGTGAATTCACTAAAATGATTCTATCAGATCGC
	Reverse	CAGCTCGAGCTCGATGGATCCGAAATCAAAAGGGTTTGGCACATT
49.77.3	Forward	GCCATGGAGGCCAGTGAATTCCCACAATACTTTCAACTTGGAAAC
	Reverse	CAGCTCGAGCTCGATGGATCCGAACTGGAAAATGTGGGGAATGAG
45.413.1	Forward	GCCATGGAGGCCAGTGAATTCATGGACTTCAATTCCATTTATGAA
	Reverse	CAGCTCGAGCTCGATGGATCCTTGACCGGATGTCGGAGGTGGAGG
2085.199.1	Forward	GCCATGGAGGCCAGTGAATTCTTAACCACCACCCCTATTGATCTA
	Reverse	CAGCTCGAGCTCGATGGATCCCCGTGGCAATGTGCTAAATTGAGA
12.180.1	Forward	GCCATGGAGGCCAGTGAATTCTTGCCTTTCACTGTTTCCGACAAT
	Reverse	CAGCTCGAGCTCGATGGATCCAGTTACAATGCATTCACAAACTTT
94.274.1	Forward	GCCATGGAGGCCAGTGAATTCGATGATGGAGGATGTGCTTTTAGG
	Reverse	CAGCTCGAGCTCGATGGATCCAGATGATCCTGTAGTGGTTGCGGT
1921.111.1	Forward	GCCATGGAGGCCAGTGAATTCGTACCCGAGTTTCAATTTGATGGA
	Reverse	CAGCTCGAGCTCGATGGATCCATCAGGATGAAAGGTGCAGTCTTG

Supplementary Notes S5. **Gene annotation of the sex-determination system associated genes of *Oithona nana***

Gene	Diamond blast NR	InterProScan domains	RPKM average [illegible]
7.623.1	No hits		~5
34.133.1	No hits		~25
39.231.1	No hits		~50
87.175.1	No hits		~60
45.363.1	No hits	PAN domain	~100
25.213.1	No hits		~475
664.74.1	No hits		~500
27.64.1	No hits		~600
41.148.1	No hits		~1200
119.249.1	putative protein LLP homolog isoform X1 [Acartia pacifica]	Learning-associated protein	~1800
On_ATP5H	ATP synthase subunit d, mitochondrial-like [Eurytemora affinis]	ATP synthase D chain, mitochondrial (ATP5H)	~7500

Supplementary Notes S6: **Structure and localisation of the Oithona nana LDPs.**

e, *i* and *m* correspond to extracellular, intracellular and membranous respectively.

Protein name	Protein size (aa)	#Total LNR domain (#canonical LNR - # LNR-like)	Other InterPro domains	Localisation
100:211670..212713	328	3 (1-2)	-	e
11.480.1:721370..723090	531	1 (0-1)	Metallo-peptidase family M12	e
12.169.2:773247..776065	808	5 (5-0)	Trypsin	e
12.175.1	386	1 (0-1)	Trypsin	e
12.182.1	445	2 (0-2)	Trypsin	e
12.185.1	357	1 (0-1)	Trypsin	e
123:42215..42819	181	2 (0-2)	-	e
1239:190..1043	228	2 (0-2)	-	e
14.373.1:814017..816142	226	2 (0-2)	-	e
1531:53..2073	222	3 (0-3)	-	e
1646:48..560	147	1 (0-1)	-	e
174:49279..49938	219	3 (0-3)	-	e
1781:78..483	113	1 (1-0)	Metallo-peptidase family M12B	e
18.220.1:802020..803354	420	1 (0-1)	Trypsin	e
188.202.1:42571..49247	547	2 (2-0)	-	e
19.334.1	365	4 (1-3)	-	e
2.84.1	195	2 (1-1)	Trypsin	e
20.537.1	353	2 (1-1)	-	e
2085.199.1	687	6 (0-6)	-	e
21.77.1:327349..328866	458	2 (1-1)	Trypsin	e
2201:1341..2883	426	3 (0-3)	-	e
On_LDP1	378	2 (1-1)	Trypsin	e
27:531099..531903	248	2 (0-2)	-	e
32.270.1:20364..22049	561	2 (0-2)	-	e
3256:80..502	140	1 (0-1)	-	e
37:34427..34747	107	1 (0-1)	-	e
3704.33.1	266	1 (0-1)	Metallo-peptidase family M12	e
3765:1004..2202	383	1 (0-1)	-	e

38:505765..507559	438	5 (0-5)	-	e
3888:228..1050	202	2 (1-1)	-	e
4.222.1	525	2 (0-2)	Metallo-peptidase family M12	e
4.25.1	90	1 (0-1)	-	e
41:85441..86498	276	2 (0-2)	-	e
42.291.1	122	1 (0-1)	-	e
45.412.1	262	1 (0-1)	Trypsin	e
45.424.1	139	1 (0-1)	-	e
4546:128..817	229	3 (0-3)	-	e
48.267.1:6982..8492	461	2 (0-2)	Trypsin	e
48.344.1	525	2 (1-1)	Metallo-peptidase family M12	e
54.120.2:255915..258850	940	7 (0-7)	-	e
55:11070..12199	357	4 (0-4)	-	e
57.114.1	159	2 (1-1)	-	e
6.168.1:380139..381062	227	1 (0-1)	Metallo-peptidase family M12B	e
68.40.1	493	1 (0-1)	Metallo-peptidase family M12	e
821:3497..4457	291	6 (0-6)	-	e
On_LDP2	116	1 (0-1)	-	e
92.33.1:248003..248641	213	2 (0-2)	-	e
98.272.1	74	1 (1-0)	-	e
99.319.1:158136..159228	344	1 (0-1)	Trypsin	e
12.180.1	489	2 (0-2)	Trypsin	i
126.234.1	823	2 (0-2)	-	i
14:255249..255707	252	1 (0-1)	-	i
15.286.1	207	2 (0-2)	-	i
1807:1535..3342	517	1 (0-1)	-	i
188:49497..50375	177	1 (1-0)	-	i
1921.111.1	137	1 (0-1)	-	i
2.15.1:36291..38976	767	13 (0-13)	-	i
2.380.1:1294082..1295071	329	2 (0-2)	-	i
20.536.3	1083	1 (0-1)	Kelch motif	i
2184:300..2200	310	1 (0-1)	Ankyrin repeat-containing domain superfamily	i
2277:24..1778	518	8 (0-8)	-	i
23.148.2	460	2 (0-2)	Metallo-peptidase family M12	i
243.135.1	316	2 (0-2)	-	i
284.43.1	132	1 (0-1)	-	i
468:255..2448	602	1 (0-1)	PAN/Apple domain ; Kelch motif	i
541.168.1	1564	7 (3-4)	-	i
556:85..5023	1494	6 (0-6)	TSP1 ; Kelch-typ_b-propeller	i
60:181372..182580	402	1 (0-1)	-	i
94.274.1:231104..233771	867	4 (0-4)	Lectin	i
147.226.2	266	3 (0-3)	-	m
36:371592..372773	336	1 (0-1)	-	m
On_Notch	2147	3(3-0)	Notch protein	m
59:239420..240555	358	2 (0-2)	-	m
76.32.1:162363..164050	435	4 (0-4)	Thrombospondin type-1 (TSP1) repeat superfamily	m
96.130.1:322..1331	262	2 (0-2)	Lectin	m

Supplementary Notes S7: **Functional annotation of O. *nana* genes over-expressed in male.**

Protein functional groups	Protein homology	Number of male-specific genes
	Allatostatin precursor protein	1
	Carboxypeptidase E	1
**Neuropeptide and hormone metabolism**	Furin-like protease	1
	Neuroendocrine convertase 2	1
	Peptidyl-alpha-hydroxyglycine alpha-amidating lyase	1
	Peptidylglycine alpha-hydroxylating monooxygenase-like	1
	ITG-like peptide	1
	Sulfoacetaldehyde acetyltransferase	1
	Total	***8***
	Bestrophin 1	1
	Excitatory amino acid transporter 1	1
**Neuropeptide and hormone transport and release**	Multiple C2 and transmembrane domain-containing protein	1
	Sodium- and chloride-dependent GABA transporter 1-like	1
	Synaptic vesicular amine transporter	1
	Synaptobrevin	1
	Synaptotagmin	1
	Synaptosomal-associated protein 25-like	1
	Neuronal calcium sensor-like	1
	Total	***9***
	FMRFamide receptor	6
	Muscarinic acetylcholine receptor	1
	GABA receptor subunit	2
	Glycine receptor subunit	2
**Neuropeptide and hormone receptors**	Ionotropic glutamate receptor subunit	2
	Capa receptor	1
	Octopamine receptor subunit	1
	Corticotropin hormone receptor	1
	Acetylcholine receptor subunit	1
	Total	***17***
	Kv channel-interacting protein 1-like	2
**Neuron polarization Current propagation and ion channel**	Potassium voltage-gated channel, Shab-related subfamily	2
	Transient receptor potential protein	1
	Two pore potassium channel protein sup-9	1
	Total	***6***
	Beta-1,4-glucuronyltransferase 1	1
**Neuron development Synapse assembly**	Microtubule-associated protein futsch-like	1
		1
	Neural/ectodermal development factor IMP-L2-like	1
	zwei Ig domain protein	2
	copine-like	1
	synaptogenesis syg-2	1
	Total	***8***
Total		***48***

Supplementary Notes S8: **Phylogenetic tree of On_IGFBP7.**

The numbers at internal branches show the bootstrap branch support (100). We used NCBI data from *Mus muculus* (mouse), *Danio rerio* (danre) and *Bos taurus* (bovin).

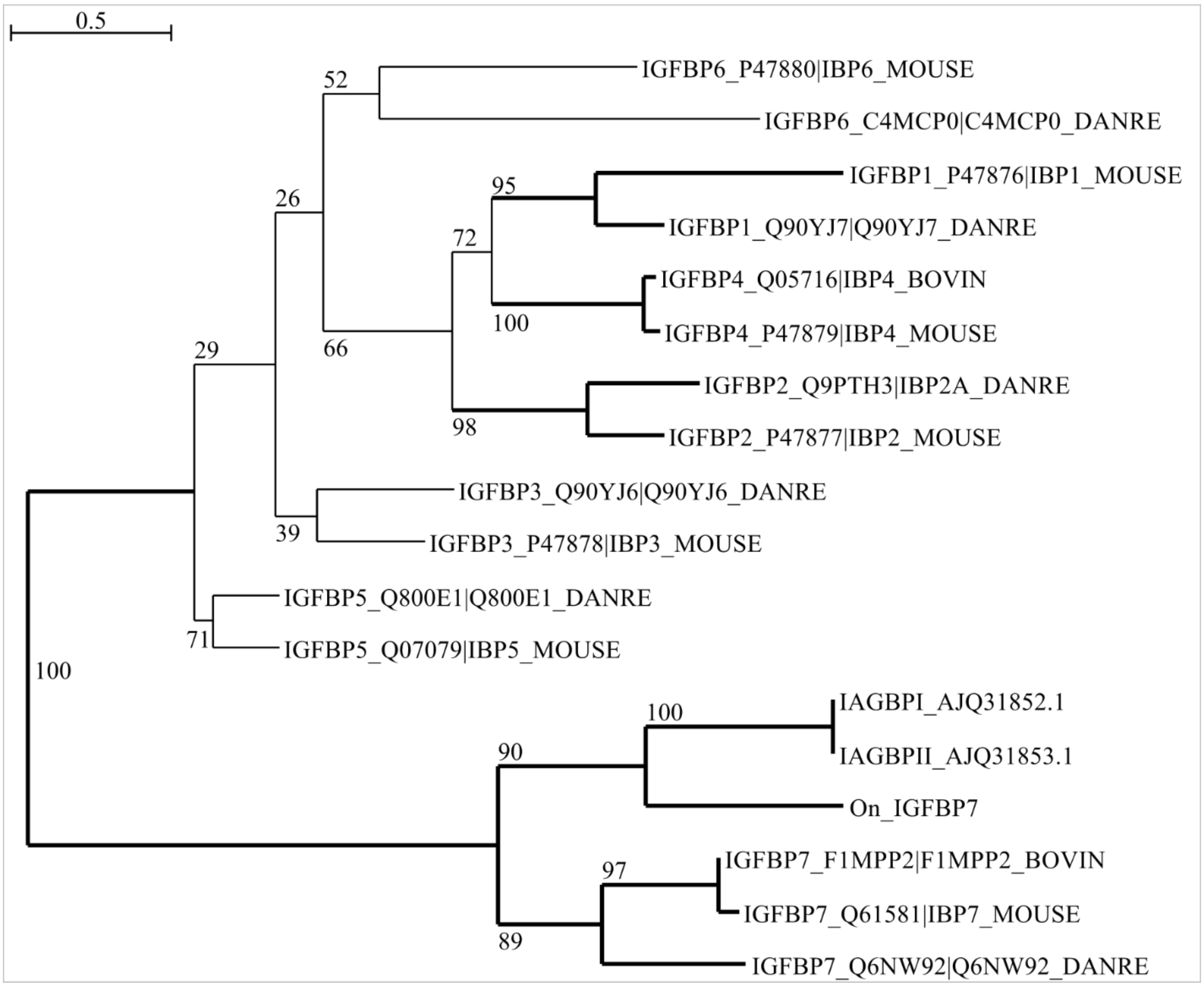

Supplement: Supplementary file 1 [file biology-10-00657-s001.zip › biology-1262359-supplementary.pdf]
